# Comparison of empirical high-dose and low-dose of meropenem in critically ill patients with sepsis and septic shock: A randomized controlled study protocol

**DOI:** 10.1097/MD.0000000000022829

**Published:** 2020-12-18

**Authors:** Xiaolin Ye, Fei Wang, Wenqing Zeng, Yueping Ding, Bin Lv

**Affiliations:** a Department of Intensive Care Unit, The Second affiliated Hospital of Zhejiang Chinese Medical University; b Department of Gastroenterology, The First affiliated Hospital of Zhejiang Chinese Medical University, Hangzhou City, Zhejiang Province, China.

**Keywords:** high dose, meropenem, protocol, sepsis, septic shock

## Abstract

**Background:**

Sepsis and septic shock syndrome are the main problems in modern medicine. Current treatment guidelines for the sepsis recommend an appropriate and timely antibiotic treatment. Meropenem has activity against a wide variety of Gramnegative and Gram-positive bacteria. At present, there are few studies on the application of high-does meropenem in the patients with sepsis and septic shock. We therefore carry out the randomized controlled research to compare the low-dose and high-dose meropenem in the critically ill sepsis and septic shock patients, and to assess the safety of the two regimens.

**Method:**

This is a prospective, single-center, and randomized research authorized through the local research ethics committee of Zhejiang Chinese Medical University (No.32198276). Sixty-four participants with a diagnosis of sepsis and septic shock are analyzed. Patients who meet the following conditions will be included:

(1) patients older than 18 years old,
(2) patients diagnosed with the sepsis and septic shock, and
(3) patients (or their relatives) who have signed a consent.

Patients with the following conditions will be excluded

(1) patients with infective endocarditis and central nervous system infection;
(2) Within 24 hours after the randomization of patients needing surgery;
(3) Within 24 hours after the randomization, patients who received extracorporeal membrane oxygenation (ECMO);
(4) Patients with known meropenem allergy.

They are assigned to 2 groups, namely, the standard-does group and high-dose group, in the standard-does group, they receive low-dose meropenem (intravenous injection of 1 g meropenem for more than 30 minutes, followed by intravenous injection of 1 g meropenem for more than three hours every 8 hours), and in the high-dose group, patients receive high-does meropenem (intravenous injection of 2 g meropenem for more than 30 minutes, and then intravenous injection of 2 grams of meropenem for more than three hours every 8 hours). The main outcomes are the modified Acute Physiology and Chronic Health Evaluation II (APACHE II) and scores of Sequential Organ Failure Assessment (SOFA). And the secondary outcomes are the 14-day mortality and 28-day mortality, the rate of microbiological cure and clinical cure, ventilator-free days, vasopressor-free days, hospital-free days and the ICU-free days, as well as safety in the two regimen groups. All analysis in our work is carried out via utilizing the software of IBM SPSS Statistics for Windows, version 20.

**Results:**

Figure 1 reveal the primary outcomes and the secondary outcomes.

**Conclusion:**

This protocol can provide a reliable evidence for the safety and effectiveness of the high-dose meropenem in the critically ill sepsis and septic shock patients.

**Trial registration number:**

researchregistry6023

## 1 Introduction

Sepsis and septic shock syndrome are the main problems in modern medicine.^[1]^ In the developed countries, the incidence rate is as high as 100 in 100 thousand people, and about 2% of the hospitalized patients suffering from sepsis during admission.^[2,3]^ Although intensive care has made great progress, involving the conduction of the evidence-based guidelines, it is still related to a significant mortality rate: one-fifth to half of septicemia patients are unable to survive, and the main cause of death is multiple organ failure.^[4]^ The present treatment guidelines for sepsis recommend an appropriate and prompt antibiotic treatment, supplemented by the fluid resuscitation, vasopressin if needed, and then provided a supportive treatment for the organ failure.^[5]^ The introduction of early hemodynamic stabilization therapy and goal-directed therapy in the first six hours can in-depth decrease the mortality in sepsis patients.

Meropenem has activity against a wide variety of Gramnegative and Gram-positive bacteria.^[6,7]^ Meropenem is still an appropriate treatment option for severe infections in the critically ill patients owing to its low toxicity and broad spectrum activity.^[8]^ At present, like other β-lactam antibiotics, meropenem has a time-dependent bactericidal activity. Jaruratanasirikul et al.^[9]^ implemented the population-wide pharmacokinetics research of meropenem in the critically ill patients, suggesting that early sepsis and septic shock may require a maximum recommended 2 g dose of the meropenem every eight hours. At present, there are few studies on the application of high-does meropenem in the patients with sepsis and septic shock. We therefore carry out the randomized controlled research to compare the low-dose and high-dose meropenem in the critically ill sepsis and septic shock patients, and to assess the safety of the two regimens.

## 2 Material and methods

### 2.1 Design

This is a prospective, single-center, and randomized research which will be conducted at the Second affiliated hospital of Zhejiang Chinese Medical University between December 2020 and December 2021. This trial was authorized through the local research ethics committee of Zhejiang Chinese Medical University (No.32198276) and then was registered in research registry (researchregistry6023). Because the meropenem regimen is considered to be a standard of conventional clinical practice in ICU, there is no intervention in the process of data collection and the analysis, thus the informed consent is not needed. However, at the time of discharge, subjects are informed of their participation in the clinical research and then the written consent is acquired.

### 2.2 Inclusion and exclusion criteria

A total of 64 participants who are diagnosed sepsis and septic shock will be analyzed. Through applying random number table, a random number is assigned to all the patients in random envelope, and all the patients are divided into low-dose group and high-dose group, there are 32 patients in each group, then allocation results are hidden. Patients who meet the following conditions will be included:

(1) patients older than 18 years old,
(2) patients diagnosed with the sepsis and septic shock, and
(3) patients (or their relatives) who have signed a consent.

Patients with the following conditions will be excluded

(1) patients with infective endocarditis and central nervous system infection;
(2) Within 24 hours after the randomization of patients needing surgery;
(3) Within 24 hours after the randomization, patients who receive extracorporeal membrane oxygenation (ECMO);
(4) Patients with known meropenem allergy.

### 2.3 Intervention

They are assigned to 2 groups, namely, the standard-does group and high-dose group, in the standard-does group, they receive low-dose meropenem (intravenous injection of 1 g meropenem for more than 30 minutes, followed by intravenous injection of 1 g meropenem for more than three hours every 8 hours), and in the high-dose group, patients receive high-does meropenem (intravenous injection of 2 g meropenem for more than 30 minutes, and then intravenous injection of 2 grams of meropenem for more than three hours every 8 hours). In these two groups, meropenem is administered through separate lumen of central venous catheter using a perfusor. Patients in the above 2 groups are treated by conventional ICU physicians during their stay in the ICU and receive a standard intensive care. The calculation of creatinine clearance (ClCr) is performed with the formula of Cockcroft. In the process of study, the dosage of meropenem can be adjusted based on the ClCr. It is noteworthy that for the multi bacterial infections, patients can take antibiotics at the same time. On the basis of the specific microbial culture of patients, the degradation should be the narrow-spectrum antibiotics. The recommended antibiotic treatment duration depends on the decision made by the team of ICU physicians.

### 2.4 Clinical endpoints

The main outcomes are the modified Acute Physiology and Chronic Health Evaluation II (APACHE II)^[10]^ and scores of Sequential Organ Failure Assessment (SOFA).^[11]^ And the secondary outcomes are the 14-day mortality and 28-day mortality, the rate of microbiological cure and clinical cure, ventilator-free days, vasopressor-free days, hospital-free days and the ICU-free days, as well as safety in the two regimen groups. The result of safety in the process of antibiotic treatment is recorded daily.

### 2.5 Statistical analysis

All data are recorded into the Microsoft Excel 2010, and then they are analyzed via applying the IBM SPSS Statistics for Windows, version 20 (IBM Corp., Armonk, NY, USA). Afterwards, all the data are described with appropriate characteristics such as mean, median, standard deviation as well as percentage. Continuous and categorical variables are analyzed using χ^2^-tests and independent *t* tests, respectively. Intention-to-treat analysis is used for the outcome assessments. When *P* value < .05, it is considered to be significant in statistics.

## 3 Results

**Figure 1:**
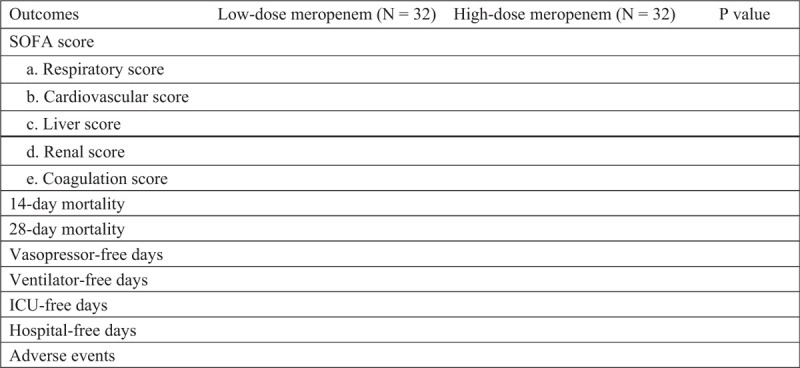
Primary and secondary outcomes.

Figure 1 reveals the primary outcomes and the secondary outcomes.

## 4 Discussion

Sepsis is considered to be a serious organ dysfunction caused by the dysfunctional host response to infection.^[12]^ Such syndrome is a significant health care issue, affecting the significant mortality. In the early stage of sepsis diagnosis, proper antibiotic treatment will help to decrease the microbial load. Therefore, the burden of microbial toxins produced or released via the bacteria will be terminated. The inflammatory response that causes tissue damage is also reduced. Because of the substantial physiological changes in the critically ill patients, the antibiotics suboptimal concentration has been reported.^[13]^ Hence, in order to acquire better clinical results, the high-dose antibiotics should be considered.^[14,15]^ Meropenem is one of the carbapenems. Due to its low toxicity and broad-spectrum activity, meropenem is extensively utilized as an empirical therapy in treating the septicemia and septic shock. Some trials have investigated the application of high doses of meropenem,^[16,17]^ but there is no sufficient evidence for the clinical efficacy of such alternative method. Although this is the first randomized controlled trial to compare different dose of meropenem in critically ill sepsis and septic shock patients, long-term of follow up with large sample size research is still required.

## 5 Conclusion

This protocol may offer a reliable basis for the effectiveness and safety of high dose in of meropenem in critically ill sepsis and septic shock patients.

## Author contributions

**Data curation:** Fei Wang.

**Investigation:** Yueping Ding.

**Methodology:** Wenqing Zeng, Bin Lv.

**Writing – original draft:** Xiaolin Ye.
